# Teachers’ Implicit Attitudes Toward Ethnic Minority Students: A Systematic Review

**DOI:** 10.3389/fpsyg.2021.712356

**Published:** 2021-09-03

**Authors:** Sara Costa, Viviana Langher, Sabine Pirchio

**Affiliations:** Department of Dynamic and Clinical Psychology, and Health Studies, Faculty of Medicine and Psychology, Sapienza University of Rome, Rome, Italy

**Keywords:** implicit attitude, ethnic bias, teacher – education, review – systematic, achievement gap

## Abstract

Although instruments to assess implicit attitudes were introduced more than 20 years ago, still there are few studies in the field of education that use them, despite the evidence that teachers with negative implicit attitudes can negatively affect the academic performance of their students. This review aims to summarize the results of studies that investigated the relationship between implicit ethnic attitudes of teachers and achievement of students. The review was conducted according to PRISMA-statement through searches in the scientific database PsychINFO, PsycARTICLES, and ERIC. Nineteen studies were included. Results show that overall teachers (from different school levels and different countries) hold negative implicit attitudes toward ethnic minority students, which play an important role in affecting the academic path of these groups of students. This review highlights the need to continue to use implicit attitudes procedures in future researches, in order to identify those factors that may contribute to the formation and expression of implicit attitudes of teachers; and the need to increase awareness of the implicit attitudes and multicultural practices of teachers in teaching programs.

## Introduction

The composition of the population of schools is profoundly changing due to the migration flows. The growing diversity could be an opportunity for both students and teachers, leading to a readjustment of teaching practices to meet the needs of students from different cultures (Passiatore et al., 2019). Unfortunately, students with an immigrant background often experience disadvantages in school, in most Organization for Economic Co-operation and Development (OECD) countries, first- and second-generation immigrant students report frequent unfair treatment from their teachers (OECD, 2019). Ethnic minority students show lower performances in school than ethnic majority peers (Haycock, 2001; Dee, 2005; Reardon and Portilla, 2015), and they drop out of school more frequently and earlier (Rumberger, 2011).

Several factors come into play when it comes to explaining this ethnic achievement gap. One of them seems to be the low socioeconomic status (SES) of ethnic minority families (Sirin, 2005), whereby ethnic minority students have a more restricted access to quality education (Strand, 2014). Further, language barriers have to be considered, since students who speak at home a language that is different from the language used in school may have disadvantages in the assessment tests used (OECD, 2016). It seems also that the levels of parental involvement and the relationship between parents and teachers are able to influence the performance of students, and the ethnic minority parents often show the lowest levels of involvement and more negative relationships with teachers (Costa et al., in press).

Teachers might influence paths of the ethnic minority students through their grading. The interactions of teachers with their students in the classroom and a judgmental bias could exacerbate the disadvantageous experience of ethnic minority students in schools. This behavior pattern of teacher can also be a consequence of the implicit attitudes and expectations that teachers have toward students and their academic paths and career opportunities (Boser et al., 2014), which are commonly negative toward marginalized groups of students, namely, ethnic minorities (Pit-ten Cate and Glock, 2019).

Studying implicit attitudes in schools is extremely important (Langher et al., 2019), as teachers are required to work in a context that implies to manage multiple tasks simultaneously (Santavirta et al., 2007) and to respond immediately to situational demands (Doyle, 2006). These conditions often do not allow teachers to engage in controlled and thoughtful processes, leaving the way open to implicit attitudes. Hence, implicit attitudes can more easily influence the behavior of teachers, their teaching practices, and their judgments about students.

Considering the aforementioned facts, the aim of this review is to provide an overview of the implicit ethnic attitudes of teachers and their relation to academic outcomes of students from ethnic minorities, investigating factors that may play a role in implicit attitudes, such as age, gender, professional status of teachers, and school level, with a focus on the different methods used to assess implicit attitudes.

## Implicit Attitudes

Attitudes represent a mental association between an attitude object and its assessment (Eagly and Chaiken, 1993). They can be toward an object, an abstract concept such as inclusion, a person, or a group (Eagly and Chaiken, 1993). Attitudes are defined as *“a psychological tendency that is expressed by evaluating a particular entity with some degree of favor or disfavor”* (Eagly and Chaiken, 1993, p. 1).

People develop attitudes throughout life, as a consequence of their own socialization processes with family and friends (Sherman, 1996; Rudman, 2004; Dovidio et al., 2010) and of their personal experiences (Sherman, 1996; Rudman, 2004). Anyhow, personal contact with the object or target group is not absolutely necessary in order to develop a set of attitudes toward them, as people can also learn from others how to evaluate these entities and they can also form their attitudes with the influence of media (Dovidio et al., 2010).

Attitudes reflect cognitive, affective, and behavioral experiences with the objects of attitude. The cognitive component of attitudes reflects socially shared knowledge and beliefs about the entity (Devine, 1989; Eagly and Chaiken, 1993) while the affective component represents the emotions and feelings associated with the attitude object (Eagly and Chaiken, 1993). The cognitive and affective components of attitudes (stereotypes and prejudices, respectively), therefore, differ in their content, since they are socially shared knowledge on the one hand and an evaluation (of a social group in our case) on the other (Eagly and Mladinic, 1989; Eagly and Chaiken, 1993), but they are often related and activated simultaneously (Eagly and Mladinic, 1989; Wittenbrink et al., 1997; Bessenoff and Sherman, 2000; Fishbein, 2008). For example, an object of attitude such as pupils of ethnic minorities, can simultaneously evoke a stereotype (the cognitive component), such as “they are bad at school,” and the evaluation (the affective component), such as “I do not like it.”

The behavioral component refers to the cognitive and affective components and represents the connection between beliefs, feelings, and (intended) behavior toward the object of the evaluation (Ajzen and Fishbein, 2005). According to this, the cognitive component can only partially predict behavior (Ajzen and Fishbein, 1980), since the behavior is composed of human beliefs, attitudes, and intentions (Ajzen and Fishbein, 2005).

When it comes to attitudes, the distinction between implicit and explicit should be considered. Implicit attitudes are automatic evaluations that come to mind in the presence of attitude object, whereas explicit attitudes are assumed to be the result of deliberative processes (Gawronski and Bodenhausen, 2006a). Hence, implicit attitudes seem to predict that automatic part of the behavior which is not subject to the intentional control (Olson and Fazio, 2009): the affective component (Fazio, 2007). On the other side, explicit attitudes reflect the cognitive component, since they are based on beliefs about the attitude object (Gawronski and Bodenhausen, 2007).

Implicit attitudes as automatic evaluations are characterized by the fact that they are uncontrolled, unaware, efficient, and unintentional (Bargh, 1994). The activation of implicit attitudes occurs as an automatic process that cannot be prevented (Devine, 1989; Bargh, 1999). The mere presence of the attitude object activates, without the need of awareness as in the case of explicit attitudes, the implicit attitude associated with it (Gawronski and Bodenhausen, 2006b).

Implicit and explicit attitudes toward a social group are often unrelated (Gawronski and Bodenhausen, 2006b). This could be due to the social desirability that comes into play in regulating the expression of explicit attitudes (De Houwer, 2006). That is, people are inclined to not show their actual attitude, but a socially accepted version of it. This is particularly true for socially sensitive issues such as the racial matter (Dovidio et al., 2009). Although implicit and explicit attitudes often do not coincide, both can have an impact on behavior (Fazio and Towles-Schwen, 1999; Olson and Fazio, 2009).

To explain the influence of attitudes on behavior, we refer to the dual-process model “Motivation and Opportunity as Determinants” (MODE) (Fazio, 1990; Fazio and Towles-Schwen, 1999). This model relies on the implicit–explicit distinction assuming that attitudes guide behavior through two different paths: explicit attitudes influence controlled and conscious behaviors, while implicit attitudes guide automatic and spontaneous behaviors. These two levels of awareness and behavior occur depending on different situations: whether people have time, cognitive resources, and motivation to reflect on their behavior and thus control it, or whether they do not have sufficient cognitive resources and thus engage in automatic behavior. However, this does not mean that implicit and explicit processes are mutually exclusive. What influences the process that will determine behavior, is the situation and the opportunity to reflect, but the behavior is often mixed, and it is assumed that the automatic parts are always included (Olson and Fazio, 2009). In addition, given the automatic character of implicit attitudes, it is likely that they are always activated unconsciously, and thus have an influence on controlled processes as well (Fazio and Towles-Schwen, 1999).

## Methods to Measure Implicit Attitudes

The distinction between implicit and explicit attitudes takes place not only at the theoretical level but also at the measurement level. Direct methods are used to measure explicit attitudes, which are generally assessed using either a Likert scale or a semantic differential (Yang and Montgomery, 2013). Semantic differentials need attitude statements to be rated on a scale between bipolar adjectives (e.g., “good”–“bad”), while Likert scales require participants to indicate how strongly they agree or disagree with a statement. With these methods, respondents are directly asked to evaluate their attitudes, and it means that they are aware of what the researcher aims to measure (Petty et al., 2008).

These methods have been criticized for several reasons. Primarily, it is argued that people may not be aware of their actual attitudes (Greenwald and Banaji, 1995). Plus, assessing social sensitive issues (e.g., racial attitudes) makes it difficult to obtain results that are not biased through social desirability, because the respondents can have control over their responses, and the risk is that real attitudes are not recorded with those methods (De Houwer, 2006), but it is more likely that self-reported data reflect social norms rather than “real” attitudes (Fazio et al., 1995).

To overcome these problems, implicit attitudes should not be measured by a direct questionnaire. For these reasons, implicit measures do not rely on direct questions, but attitude is inferred from the reactions of the subject to different tasks, mostly by measuring reaction times (Wittenbrink and Schwarz, 2007).

The most used method to assess implicit attitudes is the “Implicit Association Test” (IAT; Greenwald et al., 1998), which presents a good reliability (Schnabel et al., 2008) and validity (Nosek et al., 2005; Greenwald et al., 2009).

IAT is a computer-based reaction-time procedure, and it is based on the assumption that people assign attributes to categories more quickly the more closely they are interrelated (e.g., “ethnic majority students” and “positive”). Two different categories of objects (the target attitude and a contrast attitude, for example, “students of ethnic minority” vs. “students of ethnic majority”) and evaluation (positive vs. negative) are presented in the IAT.

Reaction time is measured in milliseconds and corresponds to the time interval between the presentation of a stimulus (e.g. a word or an image on the screen) and the response of the participant (pressing on a given keyboard key) is defined as reaction times (measured in milliseconds). Above a specific threshold (3,000 ms in the IAT), reaction times are no longer considered automatic responses, because they might reflect controlled processes (Moors and De Houwer, 2006) or momentary inattention (Greenwald et al., 1998). According to the underlying assumption, people with positive attitudes toward ethnic majority group should pair positive stimulus with the category representing ethnic majority group faster than they do with negative stimulus and that same category.

A method which is also often used is the affective priming task (APT; Fazio et al., 1986, 1995), which also relies on reaction times but not on the association between concepts. Stimuli that should automatically activate a corresponding evaluation or affect (pleasant/unpleasant) are shown. The assumption is that the evaluation is still active when people are asked to categorize the words (positive or negative) that are presented immediately afterward. The reaction time in the APT is calculated between the appearance of the adjective (positive or negative) and the pressing of the key, because it is assumed that it will be faster if the valence of the adjective corresponds to the evaluation of the target stimuli presented in the previous task. Therefore, the shorter the reaction time, the stronger the association between the attitude object and the adjective. Another, less common, method to measure implicit attitudes, which does not rely on reaction times even if it refers to a similar theoretical framework, is the “affective misattribution procedure” (AMP; Payne et al., 2005). On a computer screen, the attitude object (e.g., pictures of ethnic minority students) or neutral objects (e.g., a gray rectangle) appears. Next, a Chinese character is presented, and the subject is asked to rate the degree of pleasantness/unpleasantness using two keys on the keyboard. This procedure has proven to be a good method to assess implicit ethnic attitudes, because it is not susceptible to social desirability (Payne et al., 2005, 2008). The underlying assumption is that the evaluation elicited by the target stimulus will still be active when the Chinese character is presented immediately afterward, on which then will be displaced. Nevertheless, since AMP is based on ratings, and therefore from explicit judgments, it is more likely to be a procedure that is susceptible to faking (Schnabel et al., 2008), more than the other implicit attitude measures that rely on reaction times.

Regardless of how they are measured, attitudes are not considered stable and unchanging throughout life. Instead, they may vary depending on contexts (Eagly and Chaiken, 1993; Gawronski and Bodenhausen, 2006a). Considering attitudes as the result of mental associations, the notion of *pattern activation* (Smith, 1996) can be useful to explain how these are not the outcome of a single process, but rather of the encounter between preexisting association in memory and external stimuli. Taking the example from Barsalou (1982), the associative pattern activated by *basketball* and *gym* can include the concept of *bouncing*, and not the concept of *floating*. While, if we think of *basketball* and *water*, the association can include the concept of *floating* but not *bouncing*. This means that the term *basketball* can evoke both concepts, *bouncing* and *floating*, but it will depend on the particular context in which the *basketball* stimulus is presented, which of these will be activated. Thus, applied to attitudes, the same object can activate different associations and different automatic affective reactions depending on the context in which the object is encountered.

## Method

The review process was conducted according to the PRISMA Statement (Moher et al., 2015). The PRISMA Statement consists of a 27-item checklist and a four-phase flow diagram, which aims to guide authors in improving the reporting of systematic reviews and meta-analyses.

## Research Strategies

A systematic search of the international literature was conducted in the following electronic databases: PsycINFO, PsycARTICLES, and ERIC. The last research was conducted on February 25, 2021. No restriction of the country or school level was made. The search strategy used the keywords: [(implicit attitudes) AND (teach^∗^ OR education) AND (ethnic^∗^)]. The generality of the keywords was purposely selected to include all the categories of students identified in the literature with the term “ethnic minorities” (e.g., first- and second-generation, students with immigrant background, newcomers) and all school grades. In addition, the reference lists of identified papers were searched.

## Eligibility Criteria

To be included in the systematic review, studies had to be published in English, as the shared scientific language, in the last 10 years (2010–2020). This period was chosen because, although the IAT was introduced more than 20 years ago (Greenwald et al., 1998), studies concerning implicit attitudes of teachers only appeared in the past 10 years (Pit-ten Cate and Glock, 2019). Only studies published in scientific journals were considered, excluding doctoral dissertations, book chapters, conference proceedings, and reports. Reviews and metanalysis were also excluded. Implicit attitudes had to be measured and the participants had to be preservice or in-service teachers, therefore, studies focused only on explicit attitudes or involving peers or parents were excluded.

**FIGURE 1:**
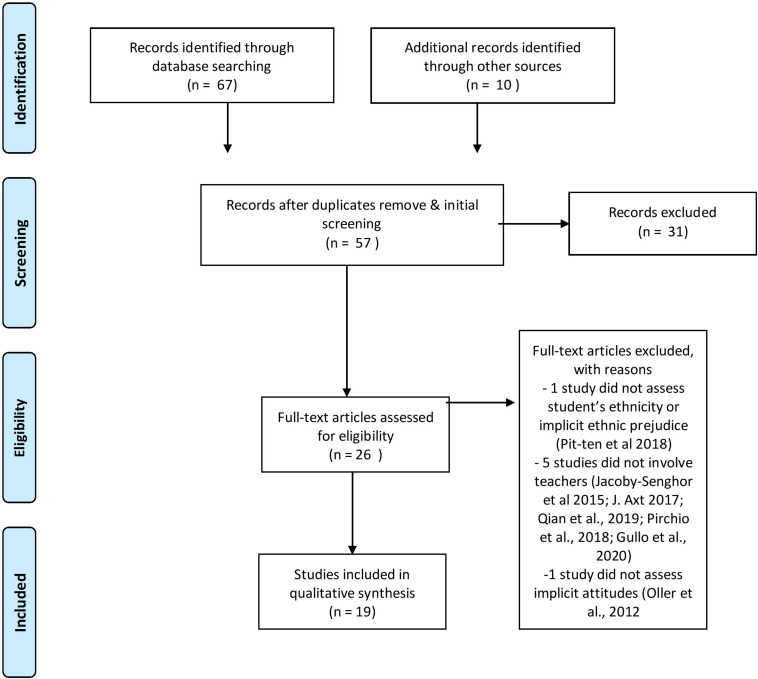
Schematic presentation of selecting studies for systematic review (in accordance with PRISMA guidelines; Moher et al., 2015).

The search identified a total of 77 articles. Mendeley reference manager software was used for removing duplicates. After removing duplicates and a first screening made by reading the title, this pool was reduced to 57 articles. Screening involved the rejection of titles if it was clearly not fulfilling the inclusion of the aforementioned criteria. After a further screening made by reading the abstract, an additional 31 articles were excluded on the basis of the same inclusion criteria. In case of uncertainty, papers inclusion was discussed and agreed upon by at least two of the three authors. The full text of 26 articles was read, leading to an exclusion of additional 7 articles (please see Figure 1 for details).

## Data Collection

According to the PICOS approach (Liberati et al., 2009), the following information has been extracted from the selected studies: authors and year of publication, country, characteristics of participants, target students, implicit methods, and materials. These data are summarized in Table 1.

**TABLE 1:** Information extracted from the selected studies.

Authorand year	Country	Sample and size	Ethnicity	Method implicit	Materials
Abacioglu et al., 2019	NL	35 primary school teachers	Majority vs. minority female	IAT	Target: student’s name (female) Attribute: words (positive/negative)
Bonefeld and Dickhäuser, 2018	DE	203 pre-service teachers	German vs. Turkish	IAT	Target: pictures (male and female) Attribute: performance-related words (good/bad)
Chin et al., 2020	United States	39,776 kindergarten to 12th grade teachers	European American vs African American.	IAT	Target: pictures (male and female) Attribute: words (good/bad)
Conaway and Bethune, 2015	United States	147 in-service tertiary teachers	Caucasian vs. Hispanic or African American	Brief IAT	Target: student name (male and female) Attribute: words (good/bad)
Glock and Böhmer, 2018	DE	63 teachers and 50 pre-service teachers	Majority vs. minority	IAT	Target: pictures (male) Attribute: words (positive/negative)
Glock and Klapproth, 2017	DE	82 primary school teachers and 82 secondary school teachers	German vs. Turkish	IAT	Target: students’ names (male) Attribute: words (positive/negative)
Glock and Kleen, 2019	DE	216 preservice teachers	German vs. Turkish	IAT	Target: student name (male) Attribute: words (positive/negative)
Glock et al., 2013	DE	40 preservice secondary school teachers	Majority vs. minority	APT	Prime: student picture (male) Attributes: words (positive/negative)
Glock et al., 2019	DE	145 preservice teachers	Majority vs. minority	IAT	Target: student name (male) Attribute: words (positive/negative)
Glock et al., 2019	DE	231 in-service teachers	Majority vs. minority	IAT	Target: student name (male) Attribute: words (positive/negative)
Glock and Karbach, 2015	DE	65 preservice teachers (different tracks)	Majority vs. minority	IAT AMP APT	Target: student picture (male) Attribute: words (positive/negative)
Harrison and Lakin, 2018a	United States	197 teachers (middle and secondary school)	Mainstream vs. English Learners	IAT	Target: words (English learner/mainstream) Attribute: words (good/bad)
Harrison and Lakin, 2018b	United States	116 preservice teachers	Mainstream vs. English Learners	IAT	Target: words (English learner/mainstream) Attribute: words (good/bad)
Kleen and Glock, 2018	DE	160 teachers	German vs. Turkish	IAT	Target: student name (male and female) Attribute:words (positive/negative)
Kleen et al., 2019	DE	149 preservice teachers	Majority vs. minority	IAT	Target: student name (male and female) Attribute: words (positive/negative)
Kumar et al., 2015	United States	241 in-service secondary school teachers	Caucasian vs. Arab/Chaldean	IAT	Target: students’ pictures Attribute: words (positive/negative)
Markova et al., 2016	DE	46 pre-service teachers	Majority vs. minority male	APT	Prime: student pictures (male) Attribute: (positive/negative)
Peterson et al., 2016	NZ	38 teachers	European vs. Asians	IAT	Target: student’s surnames Attribute: symbols of achievement (success/failure)
van den Bergh et al., 2010	NL	41 in-service primary school teachers	Dutch vs. Turkish/Moroccan	IAT	Target: student name (male) Attribute: words (good/bad)
Vezzali et al., 2012	ITA	5 primary school teachers	Italian vs. immigrant	IAT	Target: student name (male) Attribute: words (positive/negative)

## Results

Of the 19 selected articles, 15 studies were conducted in Europe, 5 in America, and 1 in New Zealand. Twelve studies were on in-service teachers (primary, middle, secondary, and tertiary), 7 studies were on preservice teachers (from different tracks), and 1 study was on both.

### Implicit Measurement Procedures

The majority of the presented studies used the “Implicit Association Test” to assess implicit attitudes (18 studies), 3 studies used the “APT,” and 1 study used the “AMP.”

In the study from Glock and Karbach (2015), three different methods of implicit measurement were used, and although they lead to different results, the conclusions that can be drawn are the same. In fact, it can be deduced that implicit attitudes of teachers toward students with migrant backgrounds are not in their favor, whether they are measured by the IAT, AMP, or APT (Glock and Karbach, 2015). More accurately, while the IAT and the AMP results showed negative attitudes of teachers toward ethnic minority students, the affective priming task revealed that teachers showed positive attitudes toward ethnic majority students rather than negative toward ethnic minority students (Glock and Karbach, 2015).

The other studies in this review that used the APT to measure implicit attitudes yielded the same results, i.e., participants showed positive implicit attitudes toward students from ethnic majority and no negative attitudes toward students with immigration background (Glock et al., 2013; Markova et al., 2016).

### Teaching Status

The studies examining attitudes of preservice teachers conclude that their implicit ethnic attitudes are negative (Bonefeld and Dickhäuser, 2018; Glock and Böhmer, 2018; Glock and Kleen, 2019; Glock et al., 2019; Kleen et al., 2019). Just one study did find positive implicit attitudes toward ethnic minority students among preservice teachers (Harrison and Lakin, 2018b).

When studies are conducted on experienced teachers, the results are the same, with the presence of implicit negative attitudes toward ethnic minority students (van den Bergh et al., 2010; Vezzali et al., 2012; Conaway and Bethune, 2015; Kumar et al., 2015; Glock and Böhmer, 2018; Harrison and Lakin, 2018a; Kleen and Glock, 2018; Glock et al., 2019; Chin et al., 2020). Just one study found a positive implicit attitude toward ethnic majority students but not a negative one toward ethnic minority students (Abacioglu et al., 2019).

### Teacher’s and School’s Characteristics

Few studies have controlled for the gender of teachers. The studies present in this review have shown that female teachers have less negative implicit attitudes (Abacioglu et al., 2019; Chin et al., 2020).

When the ethnicity of teachers is taken into account, teachers who are part of an ethnic minority group have been shown less biased attitudes toward ethnic minority students than teachers from the majority group (Glock and Kleen, 2019; Kleen et al., 2019; Chin et al., 2020).

In addition, the percentage of ethnic minority students attending the school setting of teacher also appears to matter. Teachers working in an ethnically diverse setting, with a large percentage of ethnic minority students, also showed less biased attitudes toward ethnic minority students (Glock et al., 2019; Chin et al., 2020).

With regard to the age of teachers, in the study of Glock and Böhmer (2018) with both samples of teachers, younger teachers were found to have fewer negative attitudes toward ethnic minority students than in-service teachers, while in a study only on in-service teachers, the youngest was the least biased (Conaway and Bethune, 2015).

### Teacher’s Evaluation of Students’ Academic Achievement and Behavior in Class

One study did not strictly examine attitudes of implicit teachers, but rather attitudes as a link between ethnic minority students and performance expectations (Peterson et al., 2016).

Negative implicit attitudes have been found to predict classroom behaviors and judgments of teachers (van den Bergh et al., 2010; Kumar et al., 2015; Peterson et al., 2016; Glock and Böhmer, 2018), and it is plausible to think that these are reflected in performances of students. In fact, although only a few studies have taken into account the actual outcomes of students, the evidence that is available to date shows that the implicit attitudes of teachers are related to differences in the achievement between student groups and this makes it clear that the negative attitudes of teachers can predict academic achievement of ethnic minority students (van den Bergh et al., 2010; Peterson et al., 2016; Chin et al., 2020). The behavior of teachers, when they present negative implicit ethnic attitudes, results in the choice of teaching practices that do not promote mutual respect, do not take into account the different cultures and they are also less likely to deal with interethnic conflict (Kumar et al., 2015).

In a Dutch study by van den Bergh et al. (2010), the authors examine attitudes toward students of Turkish and Moroccan heritage, as they are the least integrated ethnic minorities in the Netherlands, thus experiencing educational disadvantage. In this study, the performance of students on standardized tests was predicted by implicit attitudes of teachers, but not explicit attitudes. Ethnic minority students in classes where teachers had more negative implicit attitudes performed worse on achievement tests than ethnic minority students in classes where implicit attitudes were more positive (van den Bergh et al., 2010).

In a large-scale study in the United States a similar result emerged, that is, disparities in the performance of students from different ethnic groups were much higher where teachers reported higher levels of bias toward minority ethnic students (Chin et al., 2020).

Expectations of teachers of student academic success are also influenced by implicit attitudes. van den Bergh et al. (2010) showed that teachers with negative implicit attitudes rated their ethnic minority students as less intelligent and with less promising academic future prospects compared to ethnic majority students.

In their study on preservice teachers, Bonefeld and Dickhäuser (2018) have shown how implicit attitudes play a role in predicting teacher evaluations of students with migrant backgrounds, but their results are unexpected. In fact, the authors found that preservice teachers who implicitly associated ethnic minority individuals with good performance tended to assign lower grades to the ethnic minority student. It must be said, however, that this surprising result, which goes in the opposite direction of what they expected, may be due in part to the fact that their implicit evaluation instrument did not measure attitudes purely but, more precisely, implicit stereotypes.

Concerning the behavior of the teacher in the classroom, Kumar et al. (2015), showed that teachers who had implicit negative attitudes toward students of Arab descent were less likely to promote mutual respect among students in the classroom and consequently less likely to address cultural conflicts among students by adopting culturally adaptive practices and showed less commitment to culturally sensitive teaching.

One study also showed how prejudice reduction techniques carried out by teachers, such as the promotion of positive and inclusive intergroup attitudes and relationships between students of different ethnic and cultural backgrounds, can have a positive effect on students, who then appear more engaged (Abacioglu et al., 2019).

An interesting result emerged from an Italian study, showing a link between attitudes of teachers and attitudes of students (Vezzali et al., 2012), suggesting how ethnic majority students might be influenced by implicit attitudes of teachers and thus adopt negative behaviors toward ethnic minority students themselves. However, it should be noted that the sample for this study was extremely small (five teachers) and cannot be considered representative of the population.

## Discussion

Overall, this literature review showed that teachers and preservice teachers exhibit negative implicit attitudes toward ethnic minority students. The slightly different findings could result from the assumptions underlying the different measures used. The IAT measures the associative strength between categories and attributes, whereas the APT assess the evaluation activated after a prime is presented, following the assumption that the prime facilitates the evaluation of the adjectives presented afterward. This means that the evaluation automatically activated in the APT, in response to an item, may better relate to a single object instead of to the underlying category. For these reasons, it is possible that the IAT and APT measure two different constructs (Olson and Fazio, 2003). In addition, we should keep in mind the difference between *ingroup favoritism* and *outgroup derogation.* According to social-identity theory, people tend to prefer groups associated with the self as confirmation of their positive self-esteem (Dasgupta, 2004), so they will tend to favor their ingroup and sometimes derogate the outgroups (Tajfel, 1981; Tajfel and Turner, 1986; Turner et al., 1987). But research has shown that *ingroup favoritism* may play a stronger role than *outgroup derogation* in explaining the intergroup bias (Gaertner et al., 2006; Balliet et al., 2014) and therefore even if they are constructs on the same continuum, they remain separate.

It should be noted that, in the only study where positive implicit attitudes among preservice teachers were found, no explicit reference was made to the ethnicity of the students (Harrison and Lakin, 2018b). In fact, in the target of the implicit measure, the categories were English learner students/Mainstream students. Therefore, here ethnicity was only implied by native/non-native English speaker status and teachers may have implicitly valued the willingness to learn instead. However, this was not the case among middle and secondary teachers, whose implicit attitudes toward English learner students are not only not positive but rather, in line with other studies, they are found to be negative toward the minority group of students compared to mainstream students (Harrison and Lakin, 2018a). This is part of a more general limitation in the literature relative to the consideration of the characteristics and status of the target population of attitudes. In fact, the attitudes and expectations of teachers are investigated in the literature toward a variety of definitions of “ethnic minority students”: in some cases, they are immigrant students, in other students born in the country from immigrant parents, in other, they are proper citizens belonging to minority groups. Of course, the status of students is important in defining the potential challenges in establishing a positive relationship with the school context, in reaching satisfactory levels of academic achievement and in allowing good levels of family, school partnership (Costa et al., in press). As a consequence, the attitudes and expectation that the teachers may develop toward them as a group may be influenced by these characteristics and therefore future research could address this topic with a specific attention to this issue.

An interesting finding that emerges from this literature review is the absence of a difference in implicit attitudes between preservice and in-service teachers. Despite implicit attitudes of preservice teachers toward ethnic minority students are slightly less negative than those of in-service teachers, still they are negative. It was expected that preservice teachers would not exhibit negative attitudes, as they are more likely to have had more contact with ethnic minority people and, in line with the contact theory (Allport, 1954), such experiences should have a positive effect (Pettigrew, 1998). But it is not enough to assume that younger teachers (as preservice teachers are) may have more contact with ethnic minority people. Although it has been shown that being in a setting with a higher percentage of students from minority ethnic backgrounds can reduce implicit negative attitudes (Glock et al., 2019; Chin et al., 2020), it is not just the bare contact that matters, but the positive contact experience. Thus, there are other contact experiences, such as friendships, that might be relevant in research, that can have a positive influence on attitudes (Pettigrew et al., 2011), and that have not been addressed.

The age of teachers has been considered only in one study on in-service teachers (Conaway and Bethune, 2015), in which the youngest group was the least biased. In the other studies, it was assumed that preservice teachers were younger than in-service teachers. As true as this is in most cases, it would be appropriate not to confound the variables but to use the proper age to investigate also how generational social factors may impact implicit attitudes.

Usually, most of the teachers belong to the ethnic majority group (Gay, 2010; Marx and Moss, 2011) and generally show little concern for multicultural issues, probably due to a lack of cross-cultural interaction (Garmon, 2004; Gay, 2010). Additionally, White teachers feel less comfortable and less effective when interacting with students of ethnicities other than their own and therefore unfamiliar (Kumar and Hamer, 2012). Diversity is perceived as complicated, difficult, and overwhelming to deal with by most teachers (Dooly, 2005). The matching of student and teacher ethnicity is a topic already discussed (Monroe, 2005), and it would seem that students benefit more when teachers share their same ethnic or cultural background, because it allows them to build better relationships (Ladson-Billings, 1995). Two studies considered in this review found that teachers with an ethnic minority background had more positive implicit attitudes toward ethnic minority students than ethnic majority teachers (Glock and Kleen, 2019; Kleen et al., 2019). Interestingly, implicit attitudes were more positive when teachers shared the same ethnic background as students (Kleen et al., 2019) and not any minority ethnicity. It would seem, therefore, that it is not enough for students to have an ethnic minority teacher to have advantages, but only when the teacher and student share the same ethnic minority background the gap between majority and minority ethnic students narrow.

In any case, the cultural background is not the only aspect that teachers bring with them into the classroom. Gender seems to be another aspect on which implicit attitudes differ. In general, female teachers show a lower level of implicit (and explicit) ethnic prejudice (Abacioglu et al., 2019; Chin et al., 2020). On the other hand, the gender of the student also seems to play a role, not in terms of in-group favoritism, as no same-gender favoritism emerges (Kleen and Glock, 2018), but it appears that teachers have more positive implicit attitudes toward male students than female students in the secondary school (Glock and Klapproth, 2017; Kleen and Glock, 2018). Primary school teachers, instead, had negative implicit attitudes toward male students and more positive attitudes toward female students (Glock and Klapproth, 2017). This could be explained in light of the different focus in the different school levels. In primary school, teachers tend to build affective relationships with their students, and female teachers (who are the majority of primary teachers), have better relationships with female students (Spilt et al., 2012) than male teachers. At secondary school, on the other hand, the focus is more on performance than on affective relationships, and male students perform better in STEM^1^ subjects than female students (Brotman and Moore, 2008); therefore, responses of teachers may have been mediated by this (Glock and Klapproth, 2017).

Even if only few studies have investigated the link between implicit ethnic attitudes of teachers and the achievement of students, the results show clearly how they are involved in the academic achievement of students. Teachers with negative implicit ethnic attitudes behave differently in classroom interactions, since they are less likely to promote student respect and resolve interethnic conflicts (Kumar et al., 2015) and they have negative expectations of their academic performance (van den Bergh et al., 2010). Implicit ethnic attitudes of teachers influence their judgments of ethnic minority students, and they evaluate them as less intelligent and less with fewer future academic prospects (van den Bergh et al., 2010).

## Conclusions, Limits, and Future Directions

Teachers play a critical role in creating an environment conducive to learning, and therefore they should aim to the creation of an unprejudiced space in which ethnic minority students can feel safe and can develop a sense of belonging that support their cultural identities (Carter, 2005). This could be achieved through an awareness of their own implicit attitudes toward ethnic minority students, by practicing prejudice reduction techniques (Abacioglu et al., 2019) and by engaging in cultural responding practices in the classroom and resolving interethnic conflicts (Kumar et al., 2015; Pirchio et al., 2017). It can be concluded that implicit attitudes toward ethnic minority students are negative in teachers, despite the teaching status, grade level, and country. Nevertheless, methodologically, measures of implicit attitudes could give us preference toward majority ethnic students, that does not necessarily imply negative attitudes toward ethnic minority students. Ingroup preference and outgroup derogation are distinct phenomena (Brewer, 1999) and therefore might be studied separately.

Implicit attitudes influence many aspects of social life, such as interpersonal behavior and communication, affect, and motivation (Bargh, 1994; Greenwald and Banaji, 1995), all of which are extremely important in the school setting. This literature review highlights the need of using implicit attitudes procedures in future research and the importance of educating preservice teachers to critically reflect on their attitudes and beliefs given the potential consequences that these have on their behavior, on their expectations and judgments of ethnic minority students, and on their educational practices.

A limitation of this review is that only studies in English were considered, and this may have excluded a whole section of research with results that could confirm or disconfirm the conclusions drawn here. Moreover, only one group of students was considered, but other implicit biases could come into play (e.g., toward students with Special Education Needs or students with low SES). Therefore, future research could widen the target group and take into account the differences within students, by extending the research on implicit attitudes of teachers when students exhibit different characteristics and not just belonging to an ethnic minority.

The use of implicit measures to investigate ethnic bias in teachers is still limited but growing, and as this review highlights, it is necessary to understand the role that implicit attitudes play in the academic path of students with ethnic minority backgrounds. Future research should explore the different factors that may contribute to the formation and expression of implicit attitudes of teachers (such as school contextual factors or interethnic relationships of teachers) in order to identify strategies with the aim to reduce the negativity of implicit ethnic attitudes in teachers. Teachers were reported to find it hard to discuss sensitive topics such as racism and discrimination (Vezzali et al., 2012), and one solution could be raising awareness of possible ethnic bias in teachers and introducing courses for preservice teachers that are focused on educating about cultural differences and multicultural teaching practices. Teachers need to gain awareness of and become respectful of minority ethnic students and their families and reflect on their own implicit attitudes and biases that might have consequences on their students (Cherner et al., 2020).

Interventions among teachers might be carried out employing the most effective strategies to reduce implicit prejudice, such as exposure to counterstereotypical exemplars (where participants are exposed to exemplars that contradict the stereotype of the outgroup), intentional strategies to overcome biases (participants are instructed to implement strategies to override or suppress their biases), or identifying the self with the outgroup (where they perform tasks to reduce barriers between themselves and the outgroup) (Fitzgerald et al., 2019). Nevertheless, successful interventions directed at reducing teacher bias have already been implemented (e.g., Pirchio et al., 2019), but it still remains an expensive and risky course of action due to the uncertain outcome when it comes to implicit prejudice (Fitzgerald et al., 2019).

In any case, although teachers play a key role in the education of students, it is necessary to consider that the attitudes of other people, such as parents and classmates (de Boer et al., 2010, 2012) can influence the inclusion of all students. For this reason, not only teachers should be prepared to deal with cultural diversity but also programs aimed at the inclusion of students with an ethnic minority background that involves their peers and parents, should also be considered. Principals and the broader culture of schools must also be taken into account, since it has been shown that principals and their impact on the culture’s school environment influence the cultural practices of teachers (Quinn, 2002; Brown et al., 2019). Finally, it is necessary to place these interventions within a wider framework that includes culture and society more comprehensively, addressing structural issues, social biases with the ambition to change the culture and society outside the institutions (Fitzgerald et al., 2019). Therefore, early interventions (not just on preservice teachers but on a broader population, e.g., children in school) might be the best way to prevent the formation of ethnic biases.

## Data Availability Statement

The original contributions presented in the study are included in the article/supplementary material, further inquiries can be directed to the corresponding author/s.

## Author Contributions

SC: conceptualization of the review, literature search, and writing of the original draft, revision, and editing of the manuscript. SP: conceptualization of the review, and revision and editing of the manuscript. VL: revision and editing of the manuscript. All authors contributed to the article and approved the submitted version.

## Conflict of Interest

The authors declare that the research was conducted in the absence of any commercial or financial relationships that could be construed as a potential conflict of interest.

## Publisher’s Note

All claims expressed in this article are solely those of the authors and do not necessarily represent those of their affiliated organizations, or those of the publisher, the editors and the reviewers. Any product that may be evaluated in this article, or claim that may be made by its manufacturer, is not guaranteed or endorsed by the publisher.

## Footnotes

1 Science, technology, engineering, and mathematics (STEM).
